# Thermal Radiation Sensors Based on Ionic‐Conducting Pectin Films

**DOI:** 10.1002/advs.202509863

**Published:** 2025-07-17

**Authors:** Ezekiel Y. Hsieh, Elizabeth T. Hsiao‐Wecksler, SungWoo Nam

**Affiliations:** ^1^ Department of Mechanical Science and Engineering University of Illinois at Urbana‐Champaign Urbana IL 61801 USA; ^2^ Department of Mechanical and Aerospace Engineering University of California Irvine CA 92697 USA

**Keywords:** electronic skin, iontronic, non‐contact sensing, pectin

## Abstract

Artificial electronic skins that mimic the properties and functionality of human skin are becoming increasingly important for human‐robot interactions. One ability of human skin yet to be thoroughly imitated in electronic skins is non‐contact temperature sensing. Imitating this property will be useful for creating novel touch‐free interfaces for human‐centered robotic systems. Ionic‐conducting sensing layers made from crosslinked pectin films have recently been found to exhibit extremely high contact temperature sensitivity, several orders of magnitude greater than traditional sensors. However, pectin film sensors suffer from large baseline conductance decays during prolonged measurements, and their non‐contact thermal radiation sensing capabilities have not yet been systematically investigated. Here, substantially improved thermal radiation iontronic sensing stability is first demonstrated by implementing an alternating current configuration with the pectin films. The performance of various polymeric coatings is additionally studied for preventing dehydration in pectin films to improve prolonged iontronic sensor performance. It is then shown that the pectin film sensors exhibit non‐contact temperature sensing response that closely match analytical models for radiative heat transfer rate. Altogether, the findings demonstrate clear advances toward non‐contact temperature‐based electronic skins and touch‐free interfaces from pectin or other ionic‐conducting films.

## Graphical abstract

This work reports on ionic‐conducting thin‐film sensors made from crosslinked pectin for non‐contact perception via thermal radiation sensing. Pectin‐based sensors with improved robustness are demonstrated, exhibiting continuous functionality for multiple days rather than several hours. The improved pectin‐based arrays are then validated as thermal radiation sensors for non‐contact sensing of warm objects in ambient environments.

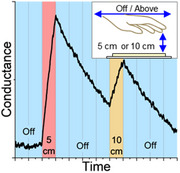

## 1 Introduction

Artificial electronic skins (e‐skins) that mimic the properties and functionality of human skin are broadly useful for applications in wearable sensing, prosthetics, and human‐robot interactions.^[^ ^1^, ^2^, ^3^ ^]^ Many e‐skin sensors and technologies have been presented over the past few decades with progressive improvements in tactile sensing and contact temperature sensing, the primary two sensory modalities of human skin.^[^ ^4^, ^5^, ^6^, ^7^, ^8^, ^9^, ^10^, ^11^, ^12^, ^13^, ^14^ ^]^ However, there has been limited development so far of e‐skin sensors that mimic or improve upon non‐contact sensory perception in human skin, which is primarily temperature‐based. Imitating this sensory modality offers unique potential for novel touch‐free interaction with human‐centered robotic systems.^[^ ^15^ ^]^

Most existing e‐skin designs for non‐contact sensing use triboelectric, capacitive, or humidity sensing mechanisms.^[^ ^15^, ^16^, ^17^, ^18^, ^19^ ^]^ However, these are each limited to only sensing objects with electrostatic, capacitive, or humid characteristics, respectively. Moreover, these designs are only capable of detecting human user input at very close ranges. For example, non‐contact sensing of user input via capacitive or humid effects has only been demonstrated under 1 cm.^[^ ^20^, ^21^, ^22^ ^]^ Although triboelectric non‐contact sensors have been demonstrated for detecting user input at increased ranges (2‐5 cm), they require the user to maintain a consistent static charge, for example, by repeatedly shuffling their feet.^[^ ^23^, ^24^ ^]^ Meanwhile, non‐contact sensing of thermal radiation commonly occurs at much greater ranges and also benefits from how the human body self‐regulates temperature. Unfortunately, most thermal radiation sensing technologies with the necessary sensitivity for non‐contact sensing require inorganic semiconductor materials and are therefore rigid and inflexible. It has thus far remained elusive to balance high thermal radiation sensitivity with the structural properties required for e‐skin applications.^[^ ^15^ ^]^

Recently, flexible ionic‐conducting sensing layers made from crosslinked pectin films have been found to exhibit extremely high temperature sensitivity, several orders of magnitude greater than traditional semiconductor‐based sensors.^[^ ^25^ ^]^ Pectin is hetero‐polysaccharide primarily made up of long galacturonic acid chains with various functional groups. Giacomo et al. found that naturally formed Ca^2+^‐pectin composites exhibited exceptionally high temperature responses and then synthesized their own artificial Ca^2+^‐crosslinked pectin films.^[^ ^25^, ^26^ ^]^ In these dried hydrogel films, Ca^2+^ ions bind to the carboxyl groups on the pectin chain, crosslinking the chains in an “egg‐box” configuration.^[^ ^27^, ^28^, ^29^ ^]^ The mobility of the Ca^2+^ ions between egg‐box sites was found to be highly temperature dependent, resulting in a temperature‐conductance relationship for the overall pectin films under applied electric potential.^[^ ^28^ ^]^ The mechanical properties and contact temperature sensitivities of these flexible pectin films have been studied in detail in prior works.^[^ ^25^, ^28^, ^29^, ^30^, ^31^ ^]^

However, there have so far not been systematic investigations of pectin film sensors for thermal radiation sensing applications, such as non‐contact sensing. This is possibly attributable to a major issue encountered when implementing pectin film sensor electrical readout. In particular, pectin film sensors exhibit a large and non‐reversible baseline conductance decay during prolonged measurements, which limits the opportunity for systematic and repeatable measurements. This becomes especially difficult when quantifying infrared (IR) sensitivity, as the temperature changes of the pectin films themselves (and the resulting changes in ionic conductance) are quite small in magnitude when compared to the baseline conductance decay. Though some have accounted for this decay with various means, particularly with extensive data normalization, we explore the specific effects of input current and encapsulation for reducing the conductance decay of pectin films. In this work, we first look into the effectiveness of an alternating current input for reducing the electrode polarization effect on baseline conductance. Next, we demonstrate the comparative effectiveness of various polymeric encapsulation coatings toward reducing water‐loss and resulting conductance decay within the films. We lastly demonstrate our pectin film sensors for thermal radiation sensing applications and validate their performance.

## 2 Results

**Figure 1:**
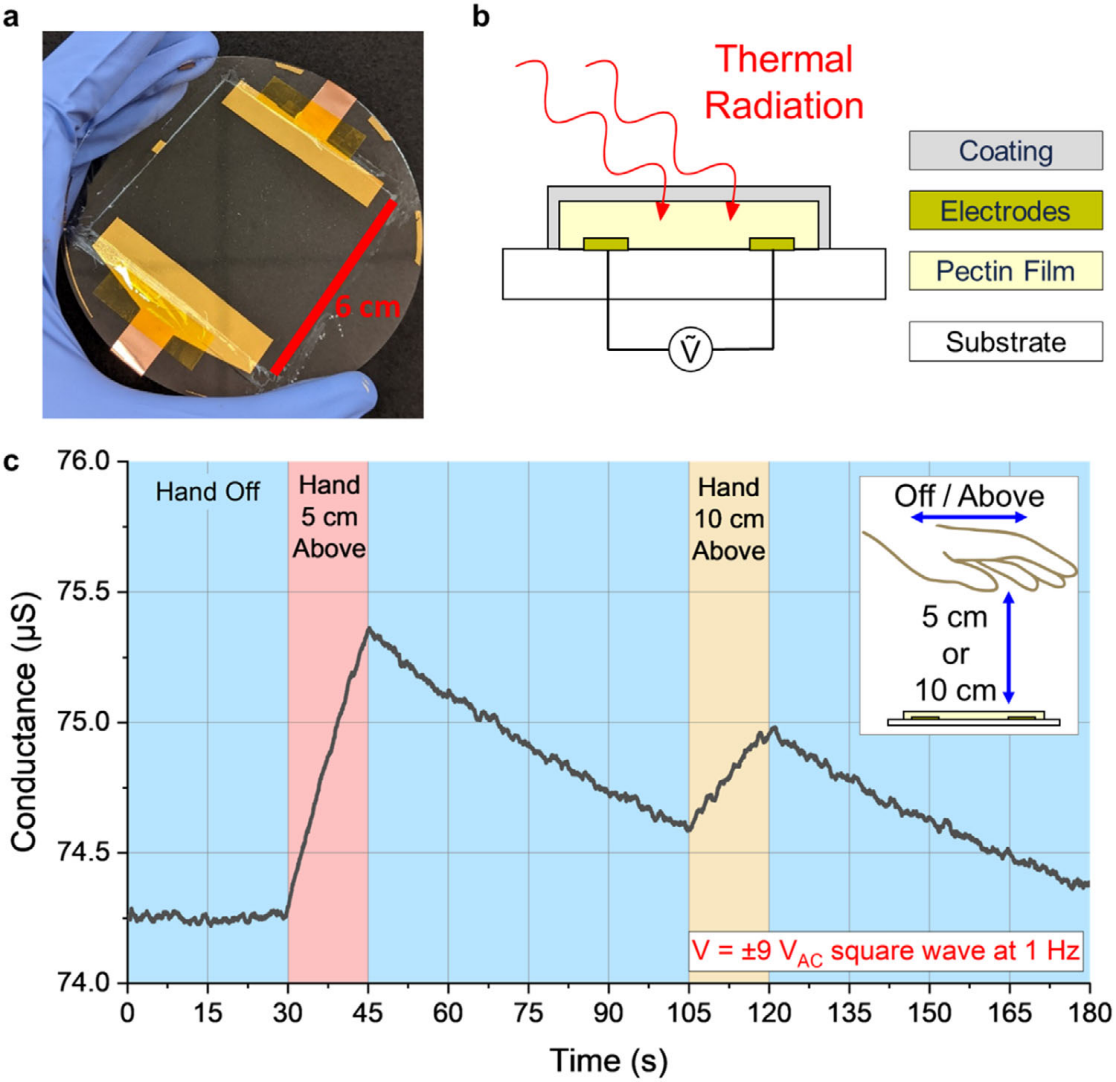
Thermal radiation sensors made from pectin films. a) Top view of sensor on glass wafer. b) Schematic of our pectin thermal radiation sensor with encapsulation layer and AC voltage input. c) Conductance of pectin film sensor under ±9 V_AC_ with hand above/off sensor at varying distances.

The general configuration of our pectin film thermal radiation sensors is shown in **Figure** **1**a,b. Each individual “pixel” consists of a pair of laterally spaced Cr/Au electrodes bridged by a drop‐casted pectin film (see experimental methods section for more details). An encapsulation layer is placed over the pectin film to protect from dehydration while a glass wafer substrate is used for handling the sensors during testing. When a voltage bias is applied across the metal electrodes, an ionic current is induced within the pectin film. This ionic conductivity is highly dependent on the temperature of the film.

A demonstration of thermal radiation sensing using our pectin pixels is shown in Figure 1c. In this demonstration, during the first 30s of ambient (≈23 °C) conditions without any input, the conductance of the pixel remains relatively constant. However, when a body‐temperature hand (≈37 °C) hovers over the pixel for 15s, the conductance begins to sharply increase, followed by a decrease back toward the baseline when the hand is removed. We attribute this conductance change to a temperature change in the pixel caused by net heat transfer from the hand to the pixel via thermal radiation and convection. The difference in positive slopes during the two hand hovering periods correlates to the difference in separation distances during those periods: 5 cm above and 10 cm above. This matches intuitively with how the radiative heat transfer rate between two objects is proportional to the inverse square of their separation distance.^[^ ^32^ ^]^ Similarly, the magnitudes of the negative slopes when the hand is removed are lower than the magnitudes of the positive slopes when the hand is hovering. This corresponds to how net radiative heat transfer is also a function of the temperature difference between two objects. The temperature difference between the hand and the pixel during heating is much greater than the temperature difference between the ambient room and the pixel during cooling. Overall, this demonstrates that the conductance of our pectin pixels varies with net radiative heat transfer, which in turn is a function of temperature difference and separation distance, offering unique potential for non‐contact sensing.

### 2.1 Input Current on Pectin Sensor Response

The simplest means for characterizing the temperature sensitivity of pectin film sensors is to apply a constant voltage drop across the films and measure the resulting ionic current. However, we observed that there is a clear decay in the baseline conductivity of pectin films when a constant DC input is applied. This was also shown in prior work, where an extended period of DC discharge was required in order to maintain a relatively stable baseline for only a few hours.^[^ ^26^ ^]^

**Figure 2:**
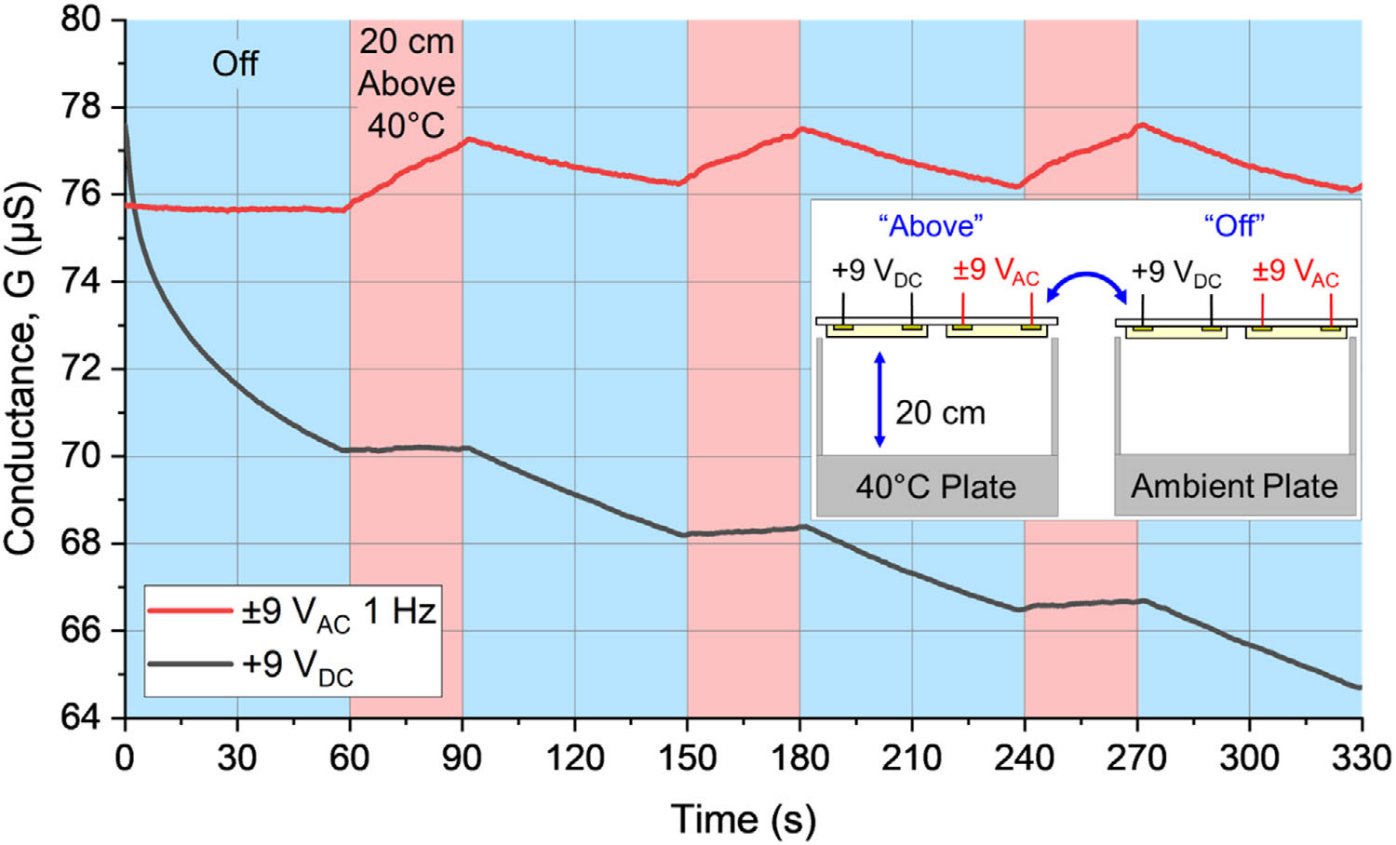
Comparison of conductance response (micro‐Siemens) of two pectin film sensors with different applied voltages, +9 V_DC_ and ±9 V_AC_, above/off a 40 °C hot plate at 20 cm.

We sought to elucidate the specific effects of input current on pectin sensor response, particularly for preventing baseline conductivity decay. In order to compare the effects of alternating and direct input voltages on pectin sensing performance, we created a 2‐pixel array on a glass handling layer shown in the **Figure** **2** inset schematic. Through one of these pixels a constant +9 V_DC_ was applied and through the other a 1 Hz ±9V_AC_ square wave was applied. The resulting ionic conductance of each pixel was measured simultaneously as described in the experimental methods section.

To place the pectin array at a consistent distance and position above a warm surface (i.e., temperature controlled hot plate), we created a stand which also served to minimize any radiative and convective thermal noise from the environment. An identical stand without heating (i.e., ambient temperature) was placed adjacent to the heated stand, so that the array could be quickly moved from being exposed to the hot plate to being exposed to ambient temperature. A description of the stand can be found in the Section Supplementary Texts and Figure S1 (Supporting Information). For a simultaneous comparison of thermal radiation sensing performance under alternating and direct input voltages, the 2‐pixel array was repeatedly placed above/off a 40 °C hot plate at a distance of 20 cm for 60s at a time, as shown in the Figure 2 inset.

The resulting conductance versus time plot for the pectin array is shown in Figure 2. When the array was placed above the hot plate, both pixels were warmed due to radiative heat transfer from the plate and the measured ionic conductance increased from the baseline. However, the baseline conductance of the two pixels varied over time. The DC pixel showed a clear baseline decrease in conductance during the entire duration of the measurement, most notably during the first 60s despite the constant ambient conditions. The shape of the conductance versus time plot for the DC pixel during 0s < t < 60s exhibited both linear and exponential decay characteristics. Meanwhile, the conductance versus time plot of the AC pixel during the same interval (first 60s) showed negligible change. The increase and decrease in conductance of the AC pixel when placed above or off the hot plate remained relatively consistent between successive intervals, demonstrating repeatable performance.

We hypothesize that the baseline decay demonstrated by the pectin pixel under applied DC voltage resulted from electrode polarization, which has been noted in other iontronic systems.^[^ ^33^, ^34^, ^35^, ^36^ ^]^ When a constant electric field is applied across an ionic‐conducting media via anode and cathode electrodes, the positively charged cations (Ca^2+^ in our pectin films) tend to collect at the cathode while the negatively charged anions (Cl^−^ in our pectin films) tend to collect at the anode. A second layer of cations and anions then are attracted to the first layers of collected anions and cations, respectively, resulting in an electric double layer (EDL). This EDL causes a large potential drop at the anode and cathode surfaces, reducing the electric field experienced within the bulk of the film. This in turn continuously reduces the apparent ionic conductivity of the pectin films when measured for a given applied electric field, as the EDL increases in thickness/density. In contrast, by oscillating the applied voltage even at low frequency (1 Hz in our experiments), a steady state can be reached where the apparent ionic conductivity of the pectin films remains relatively constant, enabling us to measure a repeatable temperature response under a constant AC voltage input. This allows for longer time‐scale measurements with more consistent responses, which are crucial for systematically investigating the thermal radiation sensitivity of our pectin pixels.

### 2.2 Encapsulation on Long‐Term Pectin Sensor Performance

During our synthesis and evaluation of pectin film sensors, we found that bare sensors tended to decrease in ionic conductivity and thermal sensitivity when exposed to ambient conditions over extended periods of time. After a few days, the baseline ionic conductivity would often drop by an order of magnitude, and after several weeks, the films sometimes fractured due to shrinkage from water‐loss which also caused increased brittleness and stress mismatch with the substrate wafer.

**Figure 3:**
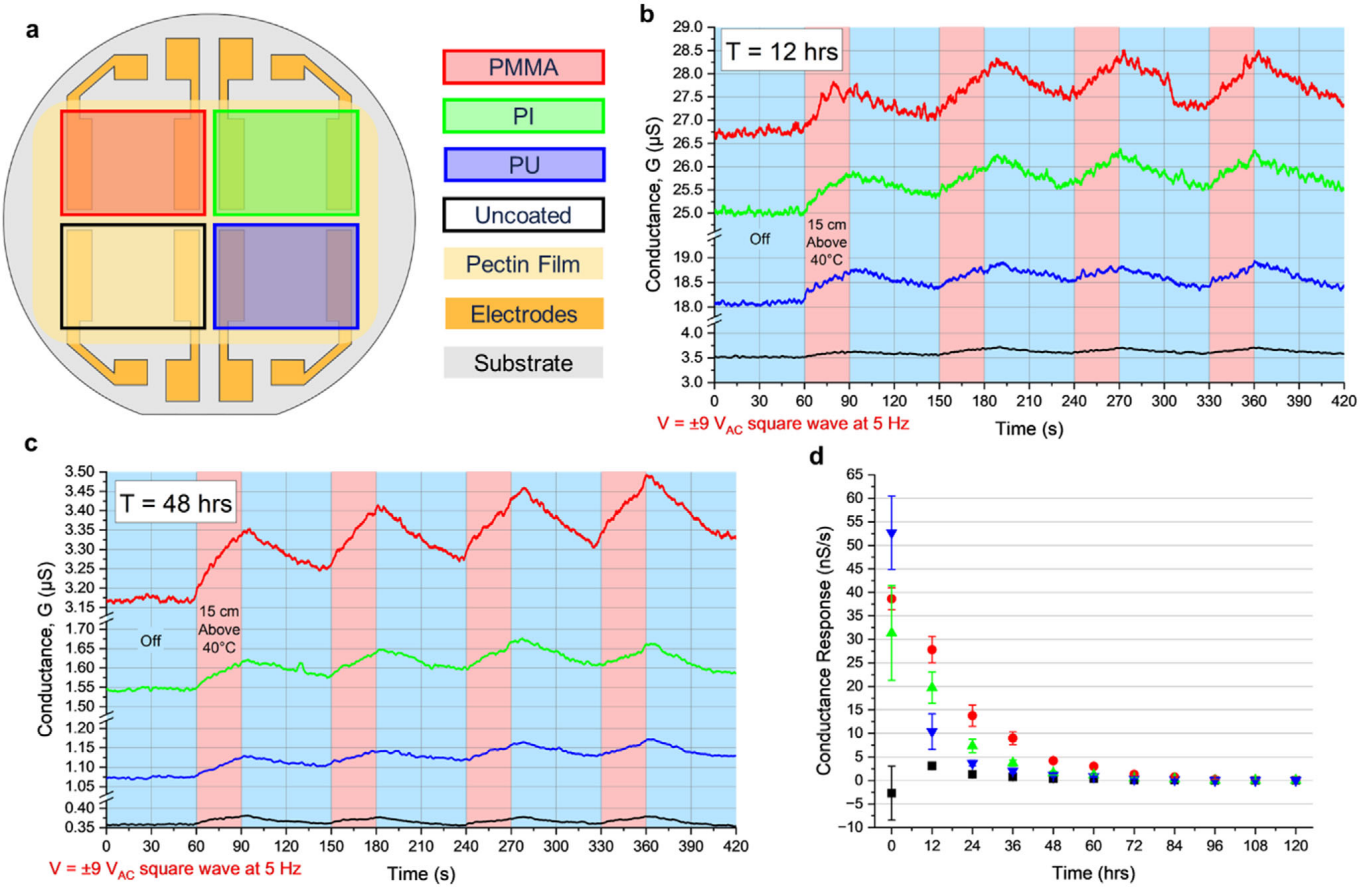
Effects of encapsulation layers to improve sensor robustness. a) Top view of 2 × 2 pectin film array with gold electrodes on a glass substrate with listed barrier coatings over each pixel (false‐colored for contrast). b) After T = 12 h of continuous operation under ±9 V_AC_, conductance of each pixel during 7‐minute experiment when placed above/off a 40 °C plate at 15 cm. c) Conductance of each pixel during identical 7‐minute experiment at T = 48 h. d) Average conductance response (nS = nanoSiemens) of all four pixels measured every 12 h. Error bars = one standard deviation.

To study the comparative effects of a few common polymeric coatings on the ionic conductivity and thermal sensitivity of our pectin sensors,^[^ ^25^, ^28^, ^29^ ^]^ we prepared a 2 × 2 pectin pixel array with three different encapsulation coatings. A schematic showing the configuration of the array is shown in **Figure** **3**a, where one pixel was spray‐coated with poly(methyl‐methacrylate) (PMMA), one pixel was covered with polyimide (PI) tape, one pixel was spray‐coated with a polyurethane (PU) coating, and the last pixel was left uncoated. All three coatings used were under 25 µm in thickness and possess high transmittance to thermal radiation in the human‐body temperature range (8‐15 µm wavelength). The coatings therefore had minimal effect on the thermal properties of the pectin array.

In order to determine the comparative effectiveness of these coatings for preventing pectin sensor degradation over time, we evaluated their performance over a 5‐day period (120 h). A 5 Hz square wave at ±9 V_AC_ was applied across each pixel for the full 120 h under ambient conditions while the conductance of all 4 pixels was measured simultaneously. Figure S2 (Supporting Information) shows the normalized conductance, G, of all four pixels over the 120‐hour measurement. Notably, the conductance of the uncoated pixel decreased by 50% within 12 h, while the conductance of the PMMA‐coated pixel did not decrease by 50% until 36 h. Additionally, the endurance times before a 90% decrease in conductance for the uncoated, PU, PI and PMMA coated pixels were ≈36 h, 48 h, 60 h, and 72 h, respectively. Every 12 h during the full 120 h experiment, a 7‐minute test was run where the sensor was placed off/above a 40 °C hot plate 4 times at a distance of 15 cm for 60s/30s, respectively.

2 of 11 resulting conductance versus time plots, taken at 12 hours and 48 hours, are shown in Figure 3b,c, respectively. The baseline conductance decay during each of the individual 7‐minute measurements was negligibly small due to the AC‐input. Additionally, all 4 pixels show some sensitivity to the 40 °C hot plate at a distance of 15 cm even after 48 h of continuous measurement. However, between 12 h and 48 h, there was a clear decrease in baseline conductance for all 4 pixels, indicated by the change in y‐axis scale.

From the plots shown in Figure 3b,c as well as the data taken at 9 other time points (not shown), we analyzed the average sensitivity of each pixel to the 40 °C hot plate at 15 cm by finding the rate of change in conductance for each pixel. The sensitivity of all 4 pixels over the full 120 h are shown in Figure 3d. The uncoated pixel exhibited the lowest sensitivity over the entirety of the experiment. The PMMA‐coated pixel showed the most stable performance where the sensitivity level decrease was the slowest among all four, followed by PI and PU. From 12 h to 48 h, the conductance response of the PMMA‐coated pixel decreased from 27.8 to 4.23 nS s^−1^, while the conductance response of the uncoated pixel decreased from 3.11 to 0.38 nS s^−1^, corresponding to an 85% decrease and an 88% decrease, respectively. All pixels showed negligibly small conductance response after 96 h of continuous testing in an ambient environment. Additional data measured using different devices comparing the sensitivities of uncoated and PMMA‐coated pectin pixels during the first 12 h of testing with more frequent measurements is shown in Figure S3 (Supporting Information). From these experiments we determined that it was critical to passivate the pectin film sensors with an encapsulation layer to preserve their sensing integrity when taking longer timescale measurements.

### 2.3 Systematic Study of Thermal Radiation Sensing Performance of Pectin Films

**Figure 4:**
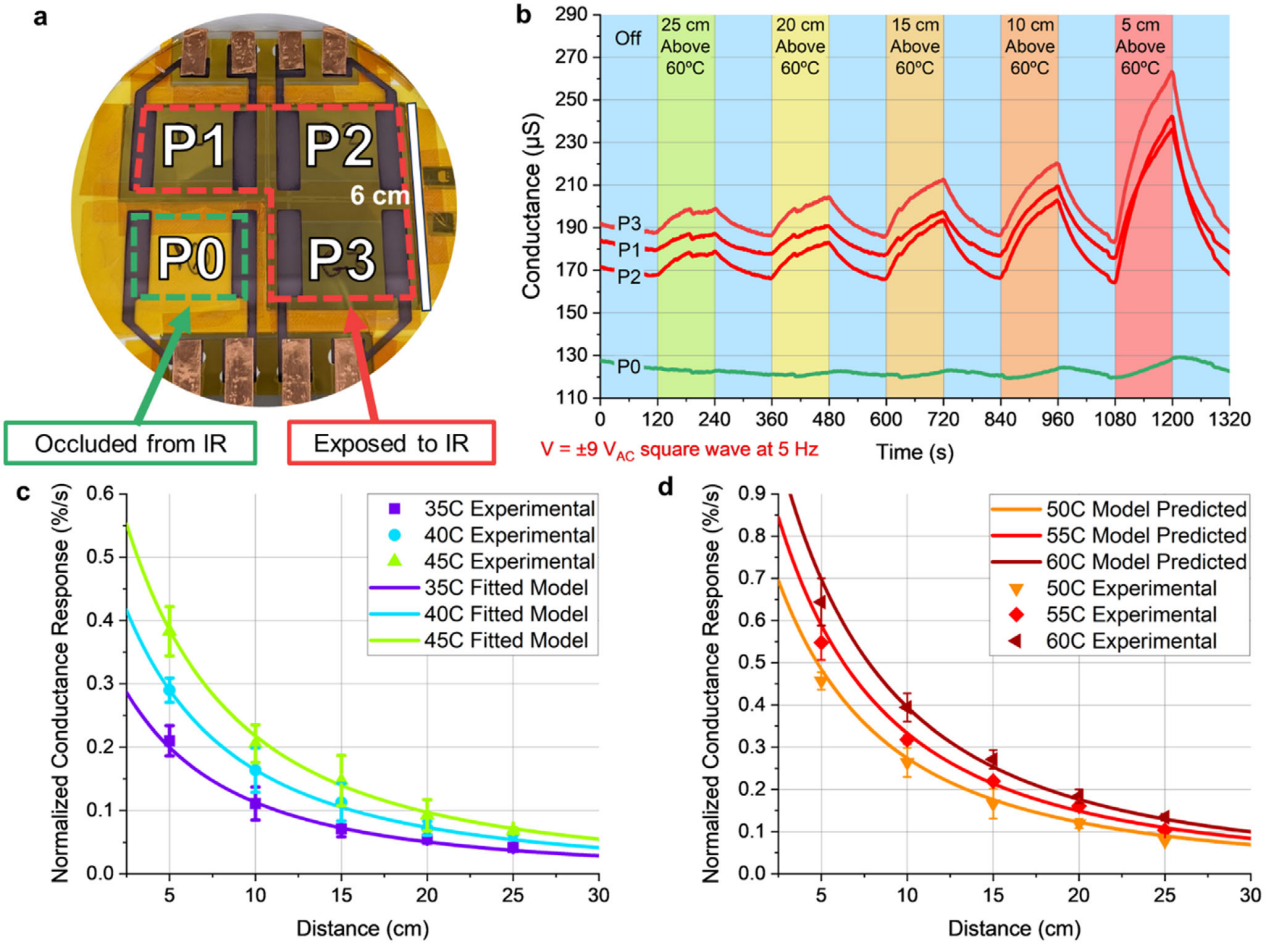
Quantifying thermal radiation sensitivity of pectin films as a function of source temperature and separation distance. a) 2 × 2 pectin array with 1 pixel occluded from incident thermal radiation and 3 pixels exposed to incident thermal radiation. b) Conductance of array under applied ±9 V_AC_ above/off a 60 °C plate at varying distances. c) Experimental values and fitted model for inverse‐square relationship between distance and normalized conductance response to thermal radiation (percentage change of ΔG/G_Ambient_ per unit time). Error bars = one standard deviation. d) Validation of fitted model for predicting normalized conductance response to thermal radiation.

In order to evaluate the thermal radiation sensing performance, we created another 2 × 2 pectin pixel array where the entire array was encapsulated with PI tape for ease of manufacturability. We occluded 1 of the 4 pixels with an IR‐reflective aluminum foil shield (P0) while the other 3 pixels were left exposed (P1, P2, P3), as shown in **Figure** **4**a. All 4 pixels experienced small but non‐negligible heat transfer due to convection and conduction. The effects of baseline heat transfer due to convection and conduction measured using P0 could then be subtracted from P1, P2, and P3 to yield the isolated effects of thermal radiation, which is explained in greater detail in the Section Supplementary Texts (Supporting Information).

We then evaluated the performance of the pectin array for thermal radiation sensing of a hot plate at ranges of 5 cm to 25 cm and hot plate temperatures of 35 °C to 60 °C, with intervals of 5 cm and 5 °C, respectively. Sample data for the measurement with hot plate temperature 60 °C are shown in Figure 4b. As expected, the IR‐occluded pixel P0 showed much smaller conductance increase when placed above the hot plate at all ranges when compared to the IR‐exposed pixels P1, P2, and P3. The non‐negligible conductance increases exhibited by P0 at closer distances to the hot‐plate can be attributed to conductive heat transfer through the sidewalls of the sensor holder and convective heat transfer from the air enclosed within the sensor holder.

The thermal radiation sensitivity of our pectin pixels can be quantified as the average normalized conductance response (ANCR, %/s), calculations for which are described in the experimental methods section. We calculated the ANCR of the three IR‐exposed pixels as a function of hot surface temperature and distance. These experimentally derived values are plotted with error bars in Figure 4c,d, where the temperature is denoted by color and the distance is denoted by the x‐axis value. For the lowest temperature surface (35 °C) at the furthest distance (25 cm) evaluated, the average normalized conductance response was 0.04%/s. Meanwhile the highest measured ANCR values exceeded 0.64%/s, indicating a nearly 10% change in conductance due to thermal radiation from a warm (60 °C) plate at 5 cm for 15 s with the sizeable convective effects already subtracted.

We then compared the experimentally obtained ANCR values with analytical models for radiative heat transfer between two surfaces. Net radiative heat transfer from a hotter surface (1) to a colder surface (2) can be expressed according to Equation 1:

(1) $$\frac{Q_{1\to 2}}{t}=\sigma e_{1}F_{1\to 2}A_{1}\left(T_{1}^{4}-T_{2}^{4}\right)$$

where *Q* is heat transfer, *t* is time, *σ* is the Stefan–Boltzmann constant (5.67 × 10^−8^ W⋅m^−2^⋅K^−4^), *e_1_* is the emissivity of the hot surface, *F_1→2_* is the view factor from the hot surface to the cold surface, *A_2_* is the area of the hot surface, and *T_1_* and *T_2_* are the temperatures of the hot and cold surfaces, respectively. For a setup with controlled parameters where only the separation distance and temperatures change, and all other parameters are kept constant, this equation can be simplified to Equation 2:

(2) $$\frac{Q_{1\to 2}}{t}\propto \frac{1}{D^{2}}\left(T_{1}^{4}-T_{2}^{4}\right)$$

where the heat transfer rate is proportional to the inverse square of the change in distance and difference of the surface temperatures to the 4^th^ power.

If we assume that the ANCR of the pectin film sensors is proportional to the rate of heat transfer into the sensors, Equations 3 and 4 can be established:

(3) $$ANCR\propto \frac{Q_{1\to 2}}{t}$$

(4) $$ANCR=a\times \frac{1}{{\left(D+b\right)}^{2}}\left(T_{1}^{4}-T_{2}^{4}\right)$$

where *a* is a constant that encompasses the proportionality, the Stefan–Boltzmann constant, the emissivity of the hot plate, the area of the hot plate, and the linear terms from the view factor equation. Meanwhile *b* is a constant that encompasses the inverse square terms from the view factor equation for our specific setup.

We then fitted values for *a* and *b* in our pectin array using the ANCR measured from P1, P2, and P3 when placed at 5 cm – 25 cm from the hot plate at 35 °C, 40 °C, and 45 °C, finding that *a* = 3.5 × 10^−12^% s^−1^ m^−2^ K^−4^ and *b* = 0.1 m. The resulting fitted model for ANCR of our 2 × 2 pectin array as a function of hot plate temperature and separation distance is shown by the colored curves in Figure 4c. As would be expected, the fitted model closely aligns to the experimental values used to generate the model.

We then applied this model to predict the ANCR for the array at 50 °C, 55 °C, and 60 °C, and compared the predictions to the experimentally measured ANCR values in Figure 4d. The model‐predicted ANCR values (curved lines) match closely to our experimentally measured values (symbols with error bars), validating our model parameters. The accuracy of our model may potentially break down when the pectin array is used to detect high temperature targets at extremely close range, but this is likely attributed to the much greater conductive and convective effects at that range, which we were unable to fully account for. Meanwhile, the model appears to be particularly accurate at ranges greater than 15 cm. Overall, the agreement between our model‐predicted thermal radiation heat transfer rates and the experimentally measured ANCRs indicates that our pectin pixels are useful for thermal radiation sensing applications in the human environment temperature range.

Extrapolating from the validated thermal radiation sensitivity model, we predicted lower limits for the thermal radiation sensitivity of our pectin sensors as shown in section Supplementary Texts (Supporting Information). Given a measurement system capable of resolving 0.01% conductance differences, we expect that our pectin pixels should be capable of detecting a 40 °C target in an ambient 23 °C environment within 1 s at up to 72 cm in range. Alternatively, we expect that our pectin pixels are capable of detecting a target only 0.6 °C warmer than an ambient 23 °C environment at a 5 cm separation distance within 1 s. Measurement systems less capable of resolving small current differences could still be used for detection but would have longer response times.

Figure S5 (Supporting Information) demonstrates the application of our pectin arrays for non‐contact thermal radiation sensing of user input. In particular, we found that our 2 × 2 pectin array could detect which pixel a user was hovering their fingers over. Although all pixels in the array showed some nonzero NCR to the input, which is expected due to the omnidirectional nature of thermal radiation, there are clear trends in the relative NCRs among the four pixels, depending on the input. In particular, the NCR was highest in the pixel directly over which the user was hovering their fingers, lowest in the pixel diagonal from the input pixel, and in‐between for the two adjacent pixels. By simultaneously reading from all pixels in a pectin array and comparing against experimentally‐determined trends, it will be possible to discern specific user input in real‐time.

## 3 Conclusion

In summary, we have developed pectin‐based thermal radiation sensing arrays and highlighted the specific effects of voltage input and encapsulation on preserving the performance of our arrays for longer timescale measurements. We have shown that by applying an alternating current input and coating the pectin films with a flexible plastic layer such as PMMA or PI, pectin arrays can function continuously for multiple days rather than several hours. We have additionally provided a clear demonstration of pectin arrays as thermal radiation sensors for non‐contact sensing of warm objects, and predicted limits for sensitivity and response time to warm targets at varying temperature and range. These results advance our understanding of the performance of pectin films and provide useful guidelines to further develop pectin‐based temperature sensors in the future. We believe the results will also be broadly useful for the development of non‐pectin ionic‐conducting temperature sensors that operate based on similar principles. Overall, this work provides systematic framework towards the development of non‐contact sensors for electronic skins made from ionic conducting materials.

## 4 Experimental Section

### Pectin Film Synthesis

Metal electrodes made from Cr/Au were evaporated onto glass wafers and then placed into empty laser‐cut molds. Next, 1.5 grams of citrus peel pectin (galacturonic acid ≥ 74.0%, methoxy groups ≥ 6.7%) as a powdered basis from Sigma‐Aldrich was dissolved in 120 mL of deionized water on an 80 °C hot plate, stirring at 800 RPM for 12 h, after which the temperature was lowered to 50 °C. Next, 0.31 g of CaCl_2_ powder (corresponding to a stoichiometric ratio R = [Ca^2+^]:2[COO^−^] = 1) from Sigma‐Aldrich was dissolved in 30 mL of water. The resulting CaCl_2_ solution was then mixed into the pectin solution with a funnel at 50 °C under gentle stirring in order to evenly mix the solutions without causing bubble formation. The resulting solution was then poured into molds and allowed to settle for 24 h under ambient conditions to gelate, after which the molds were moved into a vacuum chamber and dehydrated at 200 mmHg for 72 h, under constant 2 rpm rotation to prevent uneven drying. The resulting pectin films on glass wafers were then removed from their molds. For the 2 × 1 and 2 × 2 arrays, the pixels were electrically isolated from one another by cutting away a thin region of the pectin film between each pixel. The 2 × 2 array configuration we used, with directly addressable and electrically isolated pixels, improves upon pectin arrays from prior work^[^ ^25^ ^]^ by enabling simultaneous continuous measurements across all pixels, rather than sequential measurements with time delays. Next, any encapsulation layers were either spray‐coated or adhered to the top of the sensors to prevent film dehydration. For all of the experiments undertaken in this paper, we did not remove the films from the glass substrates.

### Electrical Characterization

For each pectin pixel, the conductance was calculated from the measured voltage drop across a 100 kΩ resistor placed in series with the pixel, under a constant DC or AC square wave input voltage. In this voltage divider configuration, the voltage was split between the 100 kΩ resistor and the pectin pixel while the current was equivalent between the two. The current through the pectin pixel was calculated from the current through the 100 kΩ resistor, while the voltage drop in the pectin pixel was calculated by subtracting the voltage drop in the 100 kΩ resistor from the input voltage. The resulting conductance of the pixel was then calculated from the current and voltage. A Keithley 2600B Sourcemeter was used to provide the input voltage while an NI USB‐6218 DAQ was used to simultaneously measure the voltage drops across each of 100 kΩ resistors in series with the pectin pixels. The input frequencies used in this manuscript were chosen for ease of data processing, but we have performed some supplemental investigations of AC input frequency on sensor performance which can be found in the Section Supplementary Texts and Figure S6 (Supporting Information).

For the 2 × 2 pectin array used for validating thermal radiation sensing performance, in order to remove the effects of convective and conductive heat transfer, the conductance (G) of the IR‐occluded P0 pixel was then subtracted from IR‐exposed pixels P1, P2, and P3. Pixel‐to‐pixel variation in thermal radiation sensitivity was normalized by the baseline ambient conductance of each pixel (i.e., ΔG/G_Ambient_). From the resulting normalized conductance data for P1, P2, and P3 (see Figure S4, Supporting Information), we found the slope during the initial 15s after the array was placed above the hotplate, and used this slope (i.e., normalized conductance response, NCR, units = %/s) as an indicator of the thermal radiation sensitivity for each pixel. The NCRs of P1, P2, and P3 were then averaged for each of the temperature and separation distance combinations to determine an ANCR value for our overall pectin array.

In order to find experimental ANCR values for the full 6 × 5 matrix of temperature and separation distances (i.e., 35 °C – 60 °C and 5 cm – 25 cm), we took the following measurements: For each temperature set point, the array was placed off/above the hot plate for 120s at a time, with the separation distance starting at 25 cm and decreasing by 5 cm with each alternating off/above cycle, for a total measurement trace length of 1320s (22 min). This was then repeated for each of the 6 temperatures. The conductances of all four pixels were measured simultaneously.

### Statistical Methods

In order to visualize continuous sensor output from a discontinuous AC square‐wave input (at 1 Hz or 5 Hz), a consistent pre‐processing procedure was applied using a MATLAB script. First, the negative side of the sensor output signal was removed. Next, the remaining sensor output signal, comprised of half‐periods of positive data, was simplified by representing each half‐period (containing between 20 and 100 points, depending on the input frequency) as a single point with magnitude equivalent to the average of the entire half‐period. For sensor output from a 5 Hz input signal, a 1 second moving average filter was lastly applied, which was not used for the 1 Hz input as it would be redundant. For sensor output from a continuous DC input, only the 1 s moving average filter was applied.

## Conflict of Interest

The authors declare no conflict of interest.

## Supporting information



Supporting Information

## Data Availability

The data that support the findings of this study are available from the corresponding author upon reasonable request.
