# Supplementary material for: Thermal Radiation Sensors Based on Ionic‐Conducting Pectin Films
Source: Adv Sci (Weinh). 2025 Jul 17;12(39):e09863. doi: 10.1002/advs.202509863 (PMC12533397; doi:10.1002/advs.202509863)
Supplement: Supplementary file 1 — Supporting Information [file ADVS-12-e09863-s001.docx]

Supporting Information

**Thermal Radiation Sensors Based on Ionic-Conducting Pectin Films**

Ezekiel Y. Hsieh, Elizabeth T. Hsiao-Wecksler, and SungWoo Nam*

**Supplementary Texts:**

**Design of Stand for Supporting Pectin Array at Set Distances from Hot Plate:**

The main purpose of the stand was to ensure that the incident thermal radiation to the pixel was highly controlled. The stand was designed by us and fabricated using fused deposition modeling (FDM) 3D printing from polylactic acid (PLA) filament. Individual sections with heights of 35 mm or 50 mm were printed separately and stacked on top of one another to vary the separation distance between the pectin sensing array and the hot plate. Schematic views of these sections are shown in Figure S1b. The sections themselves were hollow cylinders with a 0.8 mm wall thickness with four smaller hollow cylinder pillars on the corner to loosely interlock between sections. The top surfaces had cut-out regions to enable ambient-temperature air into the stand while permitting only minimal thermal radiation incident from outside to the pixels. The overall conductive thermal resistance of the stand is a sum of the thermal resistances of each section as well as the thermal contact resistances between stacked sections. Due to the thinness of the walls and minimal contact area between the sections, the overall conductive thermal resistance of the stand was quite high, particularly at further separation distances. The high thermal contact resistance (i.e., low thermal conductance) of the stand significantly reduced heat conduction from the hot plate to the pectin array.

**Accounting for Conductive and Convective Heat Transfer During Thermal Radiation Measurements:**

There were two efforts in place to minimize conductive and convective heat transfer from the hot plate to the stand. First, the stand itself was 3D-printed thin-walled PLA plastic which has low thermal conductivity, particularly in the direction normal to the layers. Second, there was a thin low density polyethylene layer 12.5 mm above the hot plate that minimally attenuated thermal radiation while highly attenuating the convective heating from the hot plate itself. However, though this reduced the conductive and convective heat transfer from the hot plate to the pectin array, the values for both were still non-zero.

In order to account for the effects of non-zero conductive and convective heat transfer from the hot plate to the pectin array, we covered one pixel with a thin aluminum foil layer. This foil layer served to occlude that pixel from experiencing radiative heat transfer from the plate while still permitting the same non-zero conductive and convective heat transfer from the hot plate that the other three pixels received. By subtracting the electrical signal of the occluded pixel from that of the three non-occluded pixels and normalizing the data, we thereby accounted for the conductive and convective heat transfer into the pectin array, leaving only the radiative heat transfer signal.

**Estimating Thermal Radiation Sensitivity Limits:**

In order to estimate thermal radiation sensitivity limits for our pectin pixels, we applied the validated model for thermal radiation sensitivity as a function of surface temperature and separation distance, where ANCR has units of %/s:

$$ANCR= a\times\frac{1}{{(D+b)}^{2}}(T_{1}^{4}-T_{2}^{4})$$

$$\mathrm{where} a=3.5 \times{10}^{-12} \%\cdot s^{-1}\cdot m^{-2}\cdot K^{-4}$$

$$\mathrm{and} b=0.1 m$$

For a measurement system capable of resolving 0.01% differences in current and with a response time of at most 1s, ANCR is 0.01. For a 23ºC ambient environment, *T_2_* = 23ºC. This leaves only *D* and *T_1_* unknown. In order to find the maximum sensitivity distance for a 40ºC target, we substitute *T_1_* = 40ºC = 313.15 K and solve to find that *D* = 0.72 m. Similarly, in order to find the minimum temperature target for a set range of 0.05 m, we substitute *D* = 0.05 m and find *T_1_* = 296.75 K = 23.6ºC, which is a temperature difference of only 0.6ºC from ambient.

**Supplemental Investigations of AC Input Frequency on Sensor Performance:**

The input frequencies used in this manuscript were chosen for ease of data processing and were not intended to be highlighted as the optimal frequencies for thermal radiation sensors based on pectin films. We primarily sought to demonstrate the improvement wrought by switching from DC input to AC input. However, we performed some supplemental investigations into how pectin sensor performance varied with input frequency. We first looked at how the average baseline conductance varied with input frequency. The input frequencies investigated (1 Hz, 2 Hz, 4, Hz, 5 Hz, 10 Hz, 20 Hz, and 40 Hz) were selected as even divisors of a 400 Hz sampling rate. When the input frequency was lower, the pectin pixels experienced a longer period of conductance dissipation before the current direction switched, due to time-varying capacitive and electrode polarization effects. When the baseline conductance was calculated from the raw data values, the average was therefore lower for lower input frequencies. Meanwhile, when the input frequency was higher, the pectin pixels had a shorter period of conductance dissipation, and the average baseline conductance value was higher. This is indicated in Figure S6a.

However, when the NCR was measured from identical user inputs (in this case, a hand hovering 5 cm from the array) at varying frequencies, we found that there was no longer a frequency correlation significant in magnitude compared to thermal noise in the user input signal itself. This is shown in Figure S6b.

**Supplementary Figures:**


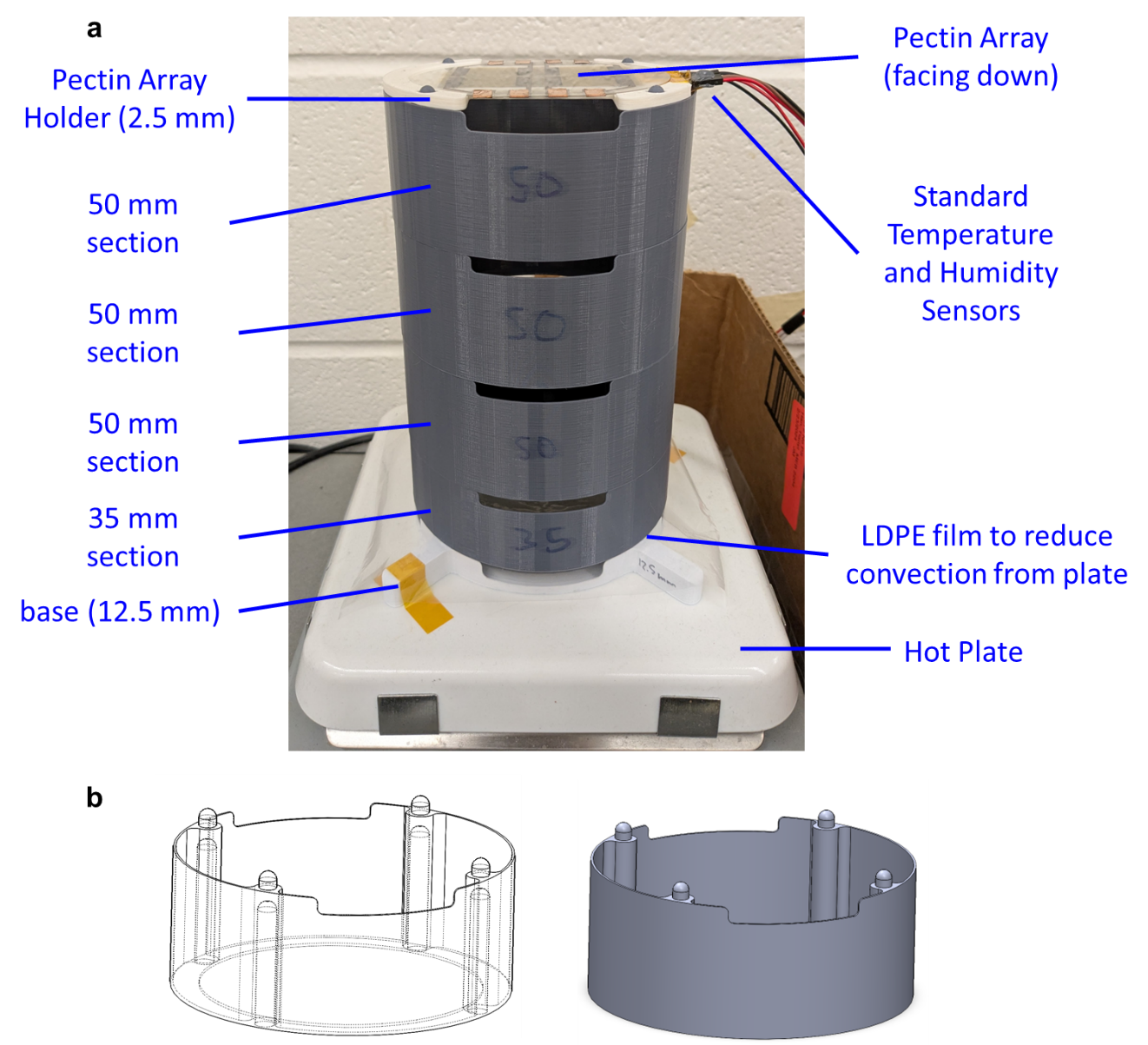


**Figure S1:** Tower Setup (a) Image of tower setup for suspending pectin array at D = 200 mm from hot plate. The separation distance can be varied incrementally by adding or removing the 50 mm sections (b) Schematic drawings of individual sections.


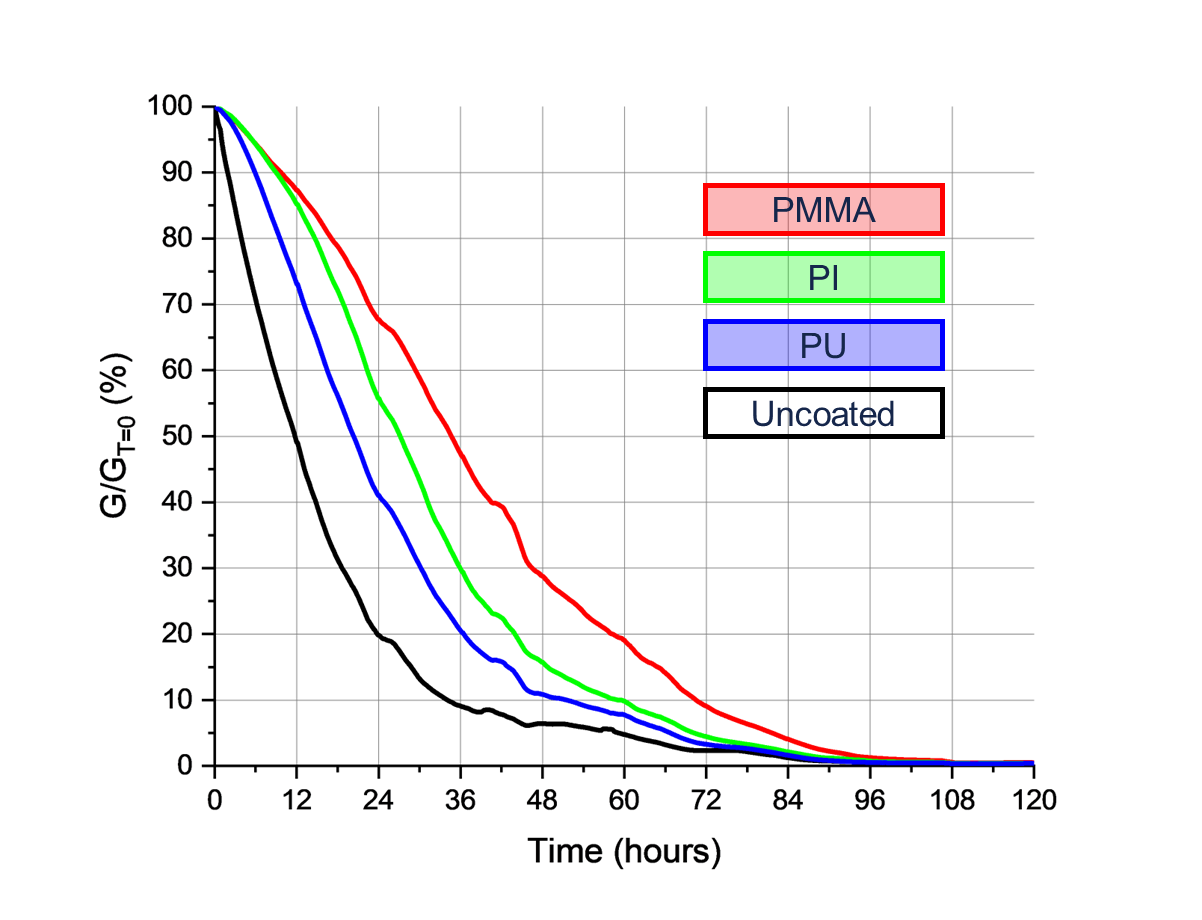


**Figure S2:** Normalized conductance (G/G_T=0_) of all four pixels in a 2x2 array with varying encapsulation coatings, under 120 hours of continuous measurement with a ±9V_DC_ 1 Hz square wave input.


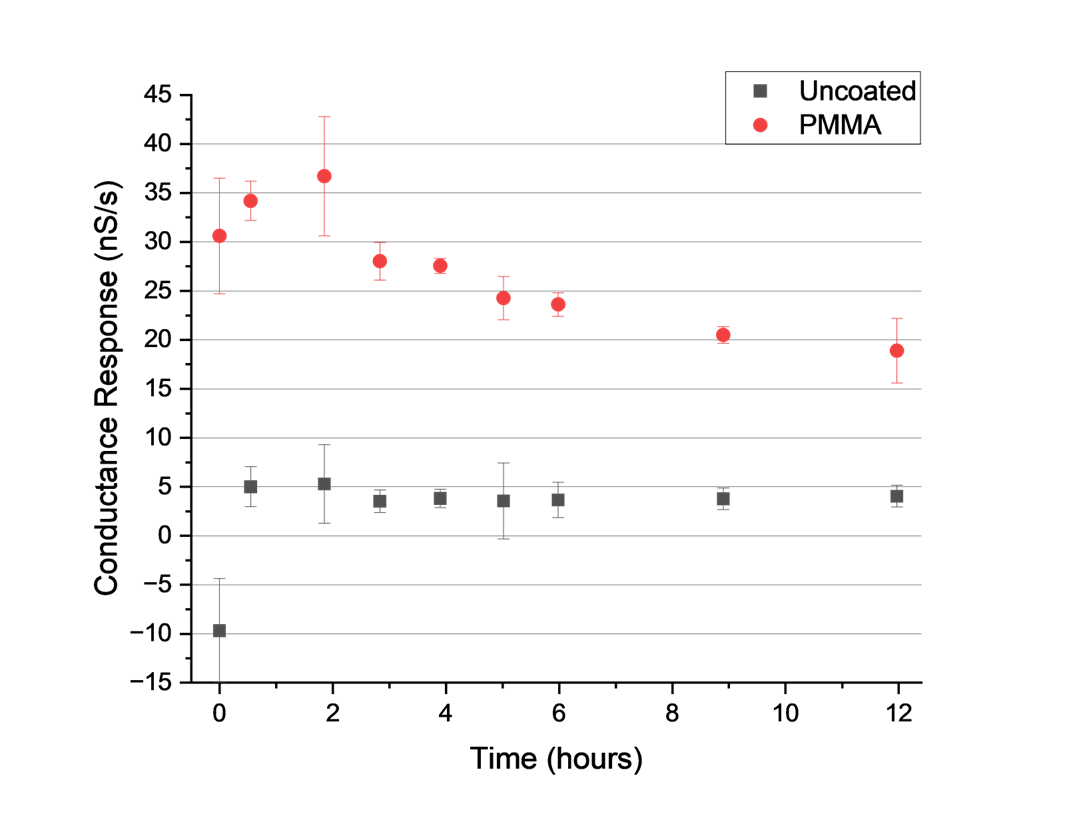


**Figure S3:** Average conductance response (nS = nanoSiemens) of uncoated and PMMA-coated pectin pixels over 18 hours of testing in an ambient environment. The conductance response was measured when a user repeatedly hovered their hand over both pixels at a range of 5 cm. The ambient environment during this experiment maintained a humidity level around 60 - 65%, in comparison to the ambient environment in Figure 3, which was maintained at a humidity level around 39 - 43%. Error bars = one standard deviation.


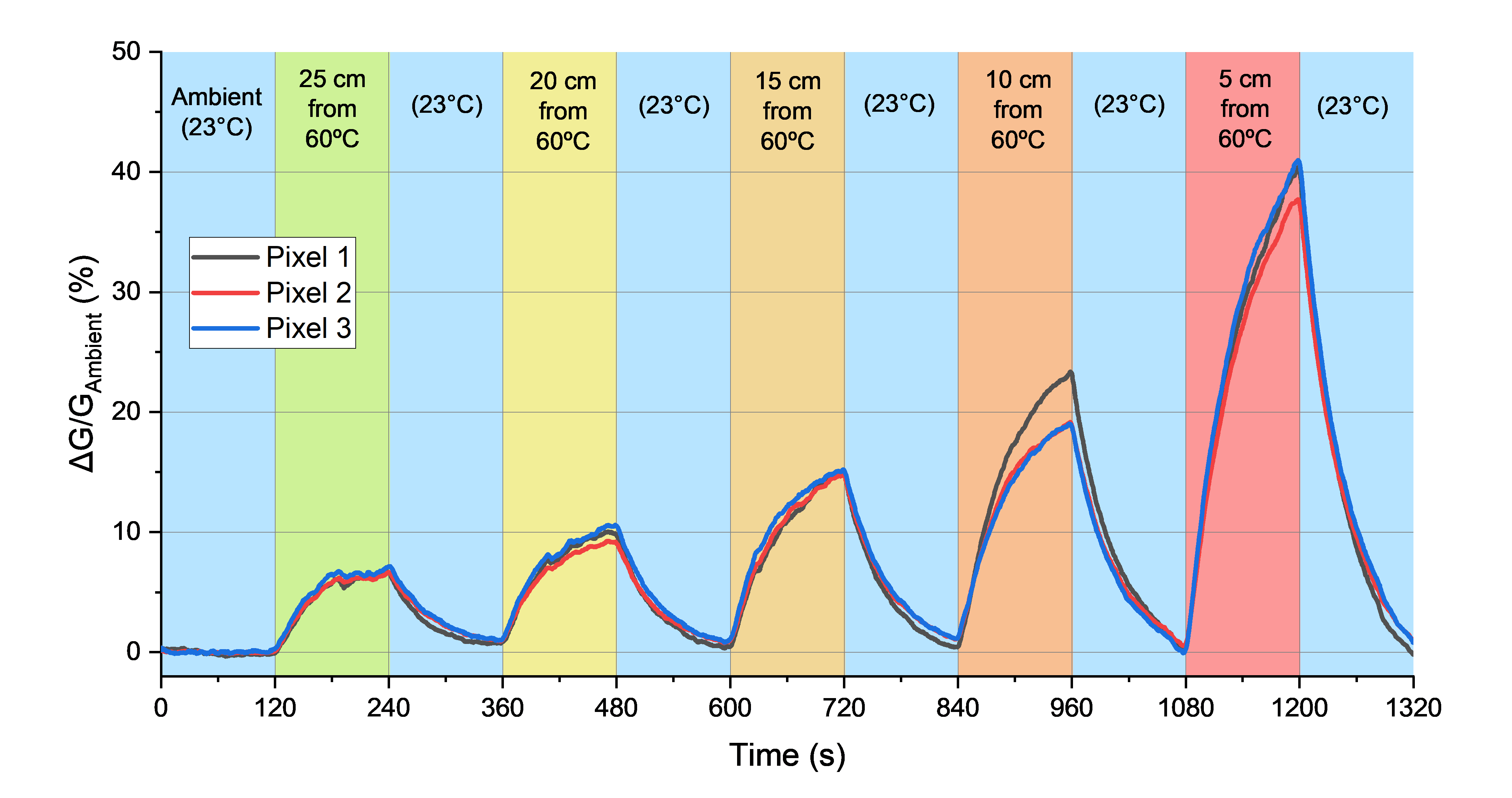


**Figure S4:** Normalized conductance response (ΔG/G_Ambient_) for the IR-exposed pixels P1, P2, and P3 calculated by subtracting the IR-occluded pixel P0 and then normalizing based on the baseline ambient conductance of each pixel.


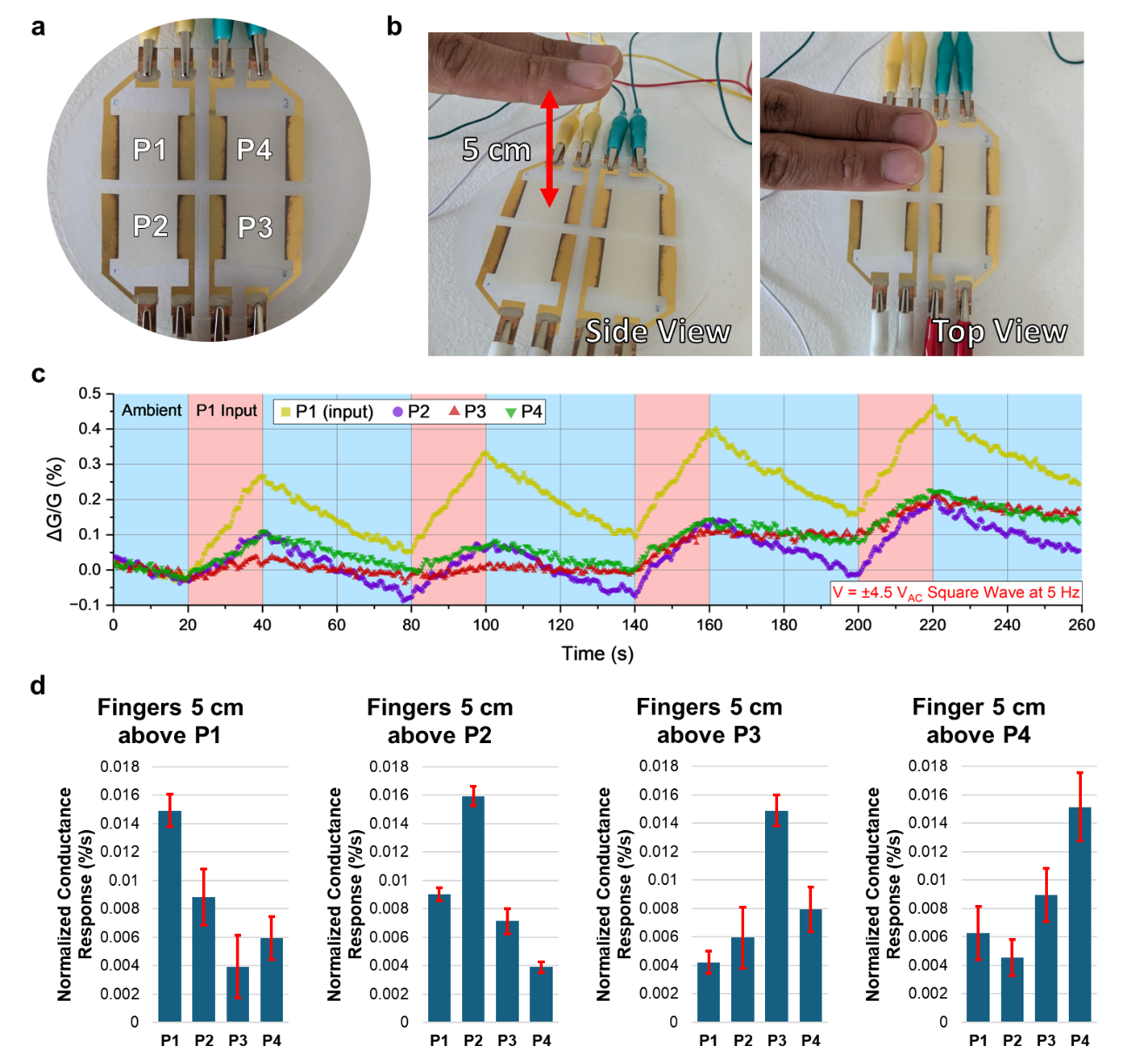


**Figure S5:** Demonstration of improved pectin array for human user input via thermal radiation emitted by their fingertips. (a) 2x2 pectin array with PMMA coating. (b) Sample images showing a user hovering their fingers 5 cm away from the array, directly above pixel 1. (c) Normalized conductance vs. time for all 4 pixels simultaneously measured as the user alternated between hovering their fingers over pixel 1 and moving their fingers away. (d) Normalized conductance response of all 4 pixels when a user hovered their fingers over each one. Error bars are the standard deviation from 4 consecutive measurements.


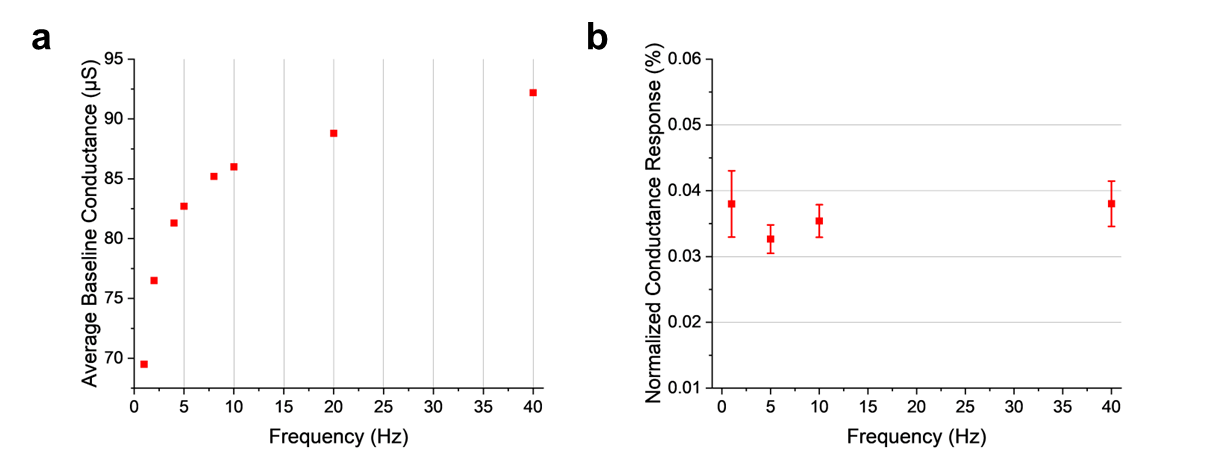


**Figure S6:** Effect of AC input frequency on sensor performance. (a) Average baseline conductance of a single pectin pixel at varying input frequency. (b) Average normalized conductance response of all four pixels in an array to a user’s repeatedly hovering their hand over the array at a distance of 5 cm in an ambient environment. Error bars are the standard deviation from 4 consecutive measurements across all 4 pixels.
